# Trends and Challenges in the Development of 3D-Printed Heart Valves and Other Cardiac Implants: A Review of Current Advances

**DOI:** 10.7759/cureus.43204

**Published:** 2023-08-09

**Authors:** Sagar Bhandari, Vikas Yadav, Aqsa Ishaq, Sailakshmn Sanipini, Chukwuyem Ekhator, Rafeef Khleif, Alee Beheshtaein, Loveleen K Jhajj, Aimen Waqar Khan, Ahmed Al Khalifa, Muhammad Arsal Naseem, Sophia B Bellegarde, Muhammad A Nadeem

**Affiliations:** 1 Cardiology, Tucson Medical Center, Tucson, USA; 2 Internal Medicine, Pt. B.D. Sharma Postgraduate Institute of Medical Sciences, Rohtak, IND; 3 Internal Medicine, Shaheed Mohtarma Benazir Bhutto Medical University, Larkana, PAK; 4 Surgery, Xavier University School of Medicine, Oranjestad, ABW; 5 Neuro-Oncology, New York Institute of Technology, College of Osteopathic Medicine, Old Westbury, USA; 6 Medicine, Xavier University School of Medicine, Aruba, ABW; 7 Internal Medicine, Xavier University School of Medicine, Chicago, USA; 8 Internal Medicine, Xavier University School of Medicine, Oranjestad, ABW; 9 Medicine, Jinnah Sindh Medical University, Karachi, PAK; 10 Medicine, College of Medicine, Sulaiman Alrajhi University, Al Bukayriyah, SAU; 11 Medicine, Mayo Hospital, Lahore, PAK; 12 Pathology and Laboratory Medicine, American University of Antigua, St. John's, ATG; 13 Medicine and Surgery, Shifa International Hospital, Islamabad, PAK

**Keywords:** review, bioprinting, valve, surgery, 3d printing, implant, cardiac

## Abstract

This article provides a comprehensive review of the current trends and challenges in the development of 3D-printed heart valves and other cardiac implants. By providing personalized solutions and pushing the limits of regenerative medicine, 3D printing technology has revolutionized the field of cardiac healthcare. The use of several organic and synthetic polymers in 3D printing heart valves is explored in this article, with emphasis on both their benefits and drawbacks. In cardiac tissue engineering, stem cells are essential, and their potential to lessen immunological rejection and thrombogenic consequences is highlighted. In the clinical applications section, the article emphasizes the importance of 3D printing in preoperative planning. Surgery results are enhanced when surgeons can visualize and assess the size and placement of implants using patient-specific anatomical models. Customized implants that are designed to match the anatomy of a particular patient reduce the likelihood of complications and enhance postoperative results. The development of physiologically active cardiac implants, made possible by 3D bioprinting, shows promise by eliminating the need for artificial valves. In conclusion, this paper highlights cutting-edge research and the promise of 3D-printed cardiac implants to improve patient outcomes and revolutionize cardiac treatment.

## Introduction and background

The development of 3D printing technology has led to notable improvements in the field of cardiology. The problems and current developments in the creation of 3D-printed heart valves and other cardiac implants are thoroughly reviewed in this article. Heart valve 3D printing trends include biocompatibility, personalization, and enhancing functional performance. In order to recreate the delicate structure and operation of human heart valves, researchers are investigating cutting-edge materials and manufacturing techniques [1]. Additionally, simulation and modeling of individual patients are being done using imaging technologies. The field of heart valve replacement therapies is evolving as new bioink formulations and cell-based methods are being investigated in order to improve tissue regeneration and lower the possibility of rejection [2].

This article examines several organic and synthetic polymers used in the 3D printing of heart valves. Natural polymers with special structural qualities, including collagen, fibrin, and chitosan, encourage tissue regeneration. Biodegradability, biocompatibility, and mechanical strength are all features of synthetic polymers such as polyglycolic acid (PGA), poly(L-lactic acid) (PLLA), poly(lactic-co-glycolic acid) (PLGA), poly(ε-caprolactone) (PCL), and polyurethane (PU) [3,4]. The manufacturing of the most suitable cardiac valves must take into account the benefits and drawbacks of each material.

Stem cells play a significant role in cardiac tissue engineering, as they can differentiate into various cell types and promote tissue regeneration [5]. This article emphasizes how pluripotent and non-embryonic stem cells may be used to lessen thrombogenic effects, reduce immunological rejection, and improve tissue availability. The exact modeling and production of 3D-printed heart valves are made possible by computer-aided design (CAD) and manufacturing (CAM), and patient-specific design employing medical imaging techniques assures compatible and tailored implants.

this article also examines contemporary developments in pacemakers (PMs) and other cardiac implants, including ventricular assist devices (VADs). Due to congestive heart failure and the scarcity of available heart transplants, VADs have emerged as crucial therapeutic alternatives [6]. PMs, which are used to correct and stabilize electrical activity in the heart, have profited from 3D printing technology since they can now be implanted specifically for each patient and have better diagnostic capabilities.

Overall, this article provides a comprehensive overview of the current trends and challenges in the development of 3D-printed heart valves and other cardiac implants. These advancements have the potential to revolutionize cardiac healthcare by offering individualized solutions, improving patient outcomes, and pushing the boundaries of regenerative medicine.

## Review

Current trends in the development of 3D-printed heart valves

3D printing has become a ground-breaking technology with enormous potential in the field of cardiac healthcare. Current trends in the development of 3D-printed cardiac valves revolve around enhancing customization, improving biocompatibility, and optimizing functional performance. In order to replicate the intricate structure and operation of real heart valves, researchers are investigating cutting-edge materials and production processes. Additionally, initiatives are being made to use imaging technology for simulation and modeling tailored to individual patients. To encourage tissue regeneration and lower the chance of rejection, novel bioink formulations and cell-based techniques are being investigated. With the help of these developments, cardiac valve replacement therapies will be transformed, offering patients with heart valve diseases individualized and long-lasting solutions [3]. Scaffolds provide the 3D framework and support for seeded cells to attach, spread, proliferate, and eventually form tissue [7]. The limited clinical translation of engineered tissues is mainly because many applications, such as heart valve and blood vessel replacements, require the construct to be mechanically functional upon implantation. Thus, understanding the functional mechanical properties of native tissues and how to mimic these properties in an engineered construct is essential (Table 1) [4].

**Table 1:** A comparison of advantages and limitations of some natural polymers in 3D printing of heart valves and other cardiac implants. PGA: polyglycolic acid, PLLA: poly(L-lactic acid), PLGA: poly(lactic-co-glycolic acid), PCL: poly(ε-caprolactone), PU: polyurethane

Material	Advantages	Limitations
Collagen	Derived from a sustainable source and requires minimal chemical processing [7].	Still far from ideal, they are expensive and potentially immunogenic and in addition they show toxic degradation and inflammatory reactions [18].
Fibrin	The degradation of the scaffold is controllable and can be adapted to the tissue development. Fibrin gel can be produced from patients’ blood and therefore the risk of immunogenic reactions are improbable [10].	Fibrin, being involved in blood clot formation, may have an increased risk of thrombosis or clotting within the valve. This can impede the proper functioning of the valve and potentially lead to serious complications.
Chitosan	Minimal foreign body reaction and water solubility depending on pH.	Low strength and inconsistent behavior with seeded cells.
PGA, PLLA & PLGA	Overall, offer a range of benefits such as biodegradability, biocompatibility, tunable degradation rates, and mechanical strength, making them valuable materials for various medical applications, including tissue engineering, drug delivery, and implantable devices.	Produce acidic byproducts, which can create an acidic microenvironment. This acidity can affect local cell behavior and tissue response, potentially leading to inflammation or adverse reactions.
PCL	It has slower biodegradation because of its relative high crystallinity and hydrophobicity. It is also easily soluble in organic solvents and has the ability to form blends.	PCL is a synthetic material that does not possess inherent cell recognition sites or bioactive cues. This can limit cell attachment and integration into the valve material.
PU	PU is flexible and elastic, allowing it to mimic the natural movement of heart valves and withstand mechanical stresses without deformation.	Despite satisfactory mechanical properties, poor biocompatibility and biostability limit the use of polyurethanes for developing an optimal PHV [19].

Natural polymers

Collagen

Collagen belongs to a naturally occurring family of proteins that is found exclusively in animals. Being a protein-based polymeric material, collagen offers unique structural properties and has the advantage of mimicking many features of the extracellular matrix. It can potentially direct the migration, growth, and organization of cells during tissue regeneration and wound healing [8]. In cardiac tissue engineering, collagen scaffolds promote cardiac commitment, vascularization, and electrical coupling. 3D collagen type-I scaffolds that have been in vitro reconstituted are one form of biomaterial that is increasingly being employed, with favorable fiber structure, mechanical qualities, biocompatibility, and biodegradability as some of its key characteristics [9].

Fibrin

Fibrin is a naturally occurring biopolymer with a variety of intriguing characteristics, including the potential to incorporate both cells and cell mediators, as well as biocompatibility, bioresorbability, simplicity of processing, and the ability to change the conditions of polymerization. The fibrin network can also be employed in autologous applications and mimics the extracellular matrix with its nanometric fibrous structure. As a result, fibrin has several uses in tissue engineering when mixed with cells, growth factors, or medications [10]. Due to its intrinsic properties, it has demonstrated superior cardiac function preservation following myocardial infarction (MI), increased cell retention, and, in some circumstances, reduced infarct sizes and improved angiogenesis [11]. Interestingly, fibrin scaffolds enhanced by thymosin 4 encapsulation functioned superiorly by increasing cell survival by almost a third, protecting against hypoxia, and enhancing heart function and wall thickness evaluated 28 days after MI [12].

Chitosan

Chitosan is a biocompatible, biodegradable, nontoxic, antimicrobial, and hydrating agent. Chitosan provides a non-protein matrix for 3D tissue growth and activates macrophages for tumoricidal activity. It stimulates cell proliferation and histo-architectural tissue organization. Chitosan is a hemostat, which helps in natural blood clotting and blocks nerve endings, reducing pain [13]. A study by Wang et al. demonstrates that chitosan scaffolds improved cardiac function and contractility, reduced infarct size and fibrotic area, and remarkably increased vessel density [14].

Synthetic polymers

*PGA*,* PLLA*, and *PLGA*

Polyesters of naturally occurring a-hydroxy acids, PGA, PLLA, and copolymers of PLGA are widely used in tissue engineering. These polymers have gained the approval of the US Food and Drug Administration for human clinical use in a variety of applications, including sutures and organ tissue synthesis [15].

Poly(ε-caprolactone)

PCL is another FDA-approved, biocompatible, and long-term biodegradable polymer with high stiffness that has been widely used in tissue engineering. One study suggests that a 3D-printed hybrid poly(glycerol sebacate), (PGS)-PCL scaffold is effective in protecting the infarcted heart against myocardial remodeling, preserving heart function, increasing LV wall thickness, reducing infarct size, promoting vascularization, inducing tissue repair by recruiting M2 macrophages, and inhibiting myocardial apoptosis [16].

Polyurethanes

Biodegradable PUs are being explored as materials for 3D-printed heart valves. They offer flexibility in design, mechanical strength, and tunable degradation rates. PU-based heart valves can gradually degrade and promote tissue growth, potentially allowing for the formation of acceptable new tissue. The first successful implantation of a mitral valve made of flexible PU with Teflon chordae tendineae in a human was performed in 1960 [17].

Stem cells

The extraordinary ability to regenerate itself exists in stem cells. During the early stages of life and growth, they can transform into a wide variety of cell types in the body. Numerous stem cell types are the subject of extensive research. “Pluripotent” stem cells (embryonic stem cells and induced pluripotent stem cells) and non-embryonic or somatic stem cells (often referred to as “adult” stem cells) are two of the primary kinds. All of the adult body's cells can be differentiated from pluripotent stem cells. The use of stem cells is a hot topic in cardiac tissue engineering because of their ability to lessen thrombogenic effects, decrease immunological rejection of grafts, and potentially makes tissues accessible on demand. Integration of cardiac fibroblasts, cardiomyocytes, and endothelial cells produced from various stem cell sources is necessary for the development of heart's architecture [20]. Adult tissues contain a stem cell population known as mesenchymal stem cells (MSCs), which can be extracted, grown in culture, and studied both in vitro and in vivo. MSCs can sustain hematopoietic or embryonic stem cells in vitro and rapidly develop into chondrocytes, adipocytes, osteocytes, etc. There is evidence to support the claim that MSCs can also express the phenotypic traits of endothelial, neuronal, smooth muscle, skeletal myoblast, and cardiac myocyte cells. MSCs can promote functional recovery when injected into infarcted hearts, but more research is still needed to fully understand how MSC differentiation occurs in cardiac scar tissue [21].

CAD and CAM

CAD is widely recognized as the key to modern design and fabrication processes, allowing designers to turn their concepts into digital representations. It also provides rapid drawing, design optimization, and virtual simulations, which provides meaningful insight into the performance/behavior of the product. CAM is used for 3D modeling, where bio-inks are printed layer by layer to form scaffolds of desired size and shape, with cells preserved inside for functional integration, maturation, and tissue formation [22].

Patient-specific design

One significant trend in 3D-printed heart valves is the focus on patient-specific design. By using medical imaging techniques such as CT scans or MRIs, researchers can create personalized models of a patient's heart to develop customized heart valves that perfectly fit their anatomy. This approach aims to improve the overall effectiveness and compatibility of the implant. The management of cardiac disease has been altered with the emergence of 3D echocardiography, and 3D modeling represents the next paradigm shift [23].

Current trends in other cardiac implants

Ventricular Assist Devices

VADs have gained prominence as one of the most frequently used surgical interventions to support the failing circulation as congestive heart failure has become an epidemic on a global scale in recent years and there are not enough heart transplants available to deal with the growing burden [24]. Since the invention of mechanical circulatory support at least 50 years ago, there have been significant advancements in the manufacturing of VADs, and these advancements have made them a preferred therapeutic choice [25]. Additionally, due to a lack of organ donors, VADs have become destination treatments as well as bridges to transplantation, acting as alternatives to cardiac transplantation [26].

In general, VADs can be classified as either right VADs (RVADs) or left VADs (LVADs), depending on which side of the heart they are implanted. However, a third type known as a biventricular assist device (BiVAD) may also be employed occasionally. This device does not really stand alone; it just refers to the simultaneous implantation of both LVAD and RVAD. Their usage is closely connected to the characteristics of heart failure. For instance, a patient with hemodynamic anomalies indicative of left heart failure, such as a cardiac index of less than 2.0 L/min/m^2^, a systolic blood pressure of less than 90 mm Hg, and a mean pulmonary capillary wedge pressure of more than 20 mm Hg, would be considered a candidate for LVAD implantation [27]. Similar to this, those who present with aberrant hemodynamic parameters that point to right heart failure, such as a cardiac index of less than 2.0 L/min/m^2^ and a right atrial or central venous pressure of greater than 20 mm Hg in the presence of a left atrial pressure of less than 10 mm Hg, are regarded as candidates for RVAD. Similarly, a combination of the hemodynamics mentioned above, including increased filling pressure and high wedge pressure together with reduced pulmonary artery saturations, points to the requirement for biventricular support in the form of a BiVAD [27].

Pacemakers

Patients with slow heart rates, symptomatic heart blocks, or heart failure may benefit from PMs to rectify the electrical activity of their hearts [28]. PMs can be classified into one of three major groups based on the number of leads or the site of attachment: single-chamber, dual-chamber, or biventricular. A single-chamber PM, like the name suggests, involves the use of a single lead that is implanted in either the upper (right atrium) or the lower (right ventricle) heart chamber. The dual-chamber type employs two leads, one for each chamber, in a similar manner. On the other hand, a biventricular PM, also known as cardiac resynchronization treatment (CRT), adds a third lead to the left ventricle to aid in its contraction when it fails to function properly.

In recent years, PM production has also benefited from the use of 3D printing technologies. Through the use of this cutting-edge approach, a flexible, specially made, implanted membrane that snugly fits on the epicardium of the heart has been created [29]. The subsequent printing of microscopic sensors on the membrane allows for the activation of the cardiac muscle in addition to the detection of a range of biochemical markers. This allows for the replacement of conventional PM leads while also offering the added benefit of minimal tissue contact with maximal diagnostic and therapeutic yield.

Clinical applications

The effective installation of cardiac devices, such as PMs, defibrillators, and heart valves, depends heavily on preoperative planning. In recent years, the development of 3D printing technology has made it a vital tool for improving preoperative planning. Surgeons may better visualize and evaluate the precise positioning and size of cardiac implants by using 3D-printed patient-specific anatomical models, which leads to improved operative outcomes [22,30].

Accurate patient-specific anatomical models may be created through 3D printing, which helps in understanding complex cardiovascular diseases. These models may be used by surgeons to plan their operations beforehand, direct their surgical technique, and improve the efficiency of their procedures [31,32]. Additionally, 3D-printed models help with patient education by allowing patients to grasp the intended surgical procedure and visualize their condition [33].

A significant development in cardiac surgery is the capacity to create customized cardiac implants. Heart valves and other cardiac implants may be made specifically for each patient's individual anatomy using 3D printing [34,35]. Due to their perfect fit, these implants lower the possibility of problems such as thrombosis, infection, and prosthetic failure [36]. Patient-specific implants also increase hemodynamic function and all-around postoperative results [37].

Tissue-engineered heart valves have a lot of potential because of 3D bioprinting, a specialized application of 3D printing. Biocompatible materials have been successfully used to print scaffolds that may be seeded with patient-derived cells to produce functional heart valve constructions [38,39]. The development of robust, physiologically active cardiac implants using this technique might surpass the need for artificial valves completely.

The effectiveness of 3D-printed models in preoperative planning for cardiac implants has been demonstrated in several studies. In a study by Valverde et al., the 3D cardiac architecture was acquired and segmented using CT and MRI. Models were created using PU filament fused deposition modeling, and their dimensions were matched to medical images. With precise representations of the cardiovascular system, 3D models help people comprehend complicated CHD. The 3D models assisted in redefining the surgical approach for 19 of the 40 selected complex cases [31]. In research by Batteux et al., the benefits of 3D technology have also been demonstrated for characterizing the architecture of arteries and directing surgical approaches. 3D models were demonstrated to be useful for the planification of intracardiac repair for complicated intracardiac anatomy [40]. Surgeons can practice difficult procedures using simulation models created through 3D printing, which improves their technical proficiency and surgical accuracy. In order to provide the best preoperative planning and lower surgical risks, surgeons might mimic valve replacement or repair procedures [41].

Limitations and challenges

Printing functional tissues has made significant strides since its inception and, as a result, is interesting enough to warrant more research. However, it is evident from the literature that, in terms of biological and technological needs, 3D printing technology is still in its infancy. In order to provide safe, efficient, and long-lasting solutions for patients with cardiovascular illnesses, researchers and engineers must address a variety of issues that have surfaced during this journey. These challenges encompass various aspects, including biocompatibility, mechanical properties, functional design, long-term performance, scalability, cost, and regulatory approval.

One of the primary issues is ensuring the biocompatibility of heart valves produced through 3D printing. It is crucial to select components for these valves that work well with the human body and do not cause an unfavorable immunological reaction that could escalate to rejection of the implant. The material also has to be able to integrate with the surrounding tissues to aid in healing and long-term stability. Extensive study and testing are required to develop appropriate biocompatible materials that can withstand the dynamic environment of the cardiovascular system. When constructing 3D-printed heart valves, durability and biodegradability are key considerations. In the body, these valves must function reliably and last for a long time. Considerations including wear resistance, degradation, and calcification must be carefully taken into account in order to ensure that the valves continue to operate optimally over time. Additionally, substances and byproducts of degradation must not be hazardous to the body when consumed. Components created through degradation, specifically in the case of the natural scaffolds detailed here, are biocompatible and naturally absorbable.

The development of affordable and scalable 3D-printed heart valves presents additional challenges. To make these valves accessible to a wider patient population, the production processes must be scalable. This calls for optimizing manufacturing processes, selecting materials that are affordable, and optimizing printing methods. To ensure that 3D-printed heart valves can be produced and delivered at a reasonable cost, it is also crucial to balance the costs of materials, tools, testing, and quality control. A critical step in the development of any medical device, including 3D-printed heart valves, is receiving regulatory approval. Meeting the strict security and efficacy requirements of the regulatory authorities requires a lot of time and effort. Extensive preclinical research, including biocompatibility tests and mechanical evaluations, is followed by rigorous clinical trials to demonstrate the safety and effectiveness of the valves in human patients. The widespread usage of 3D-printed heart valves depends on regulatory standards being met and authorization being acquired.

3D printing is a complex process involving the assembly of living or non-living structures from source image datasets to organize a model useful for application in biological studies [42,43]. In the context of cardiac implants, this is a technique that allows the fabrication of individualized structural models for patients with varied anatomic and physiologic arrangements to better facilitate pre-surgical planning and device placements. This is especially useful for patients with CHD who are set to undergo VAD placement. While the idea of 3D printing continues to gain traction, it is not devoid of challenges. The major deterrents of note are difficulties in achieving biomimicry, vascularization, and anatomically relevant biologic structures [19,42]. Because 3D printing, also known as rapid prototyping, requires a standard image dataset, to begin with, the acquisition of images displaying good blood-to-myocardium perfusion is necessary. Such images, if obtained from a CT scan, usually necessitate the use of nephrotoxic dyes, while MRI scans not only take longer but also require sedation for younger patients. Other problems, while largely technical (i.e., cost of printing, access to software, skilled personnel, etc.), continue to remain the main hurdles to its widespread use [43].

Future directions

The creation of novel materials with the required biocompatibility, mechanical strength, and longevity for cardiac implants is required as 3D printing technology develops. To improve the performance and biointegration of 3D-printed cardiac implants, more studies investigating the use of biodegradable polymers, bioinks, and bioresorbable materials are required [2,44]. A potential area for the creation of cardiac implants is the capacity to print intricate structures made of several materials or composites. It is feasible to emulate the heterogeneous character of native heart tissues and produce implants with greater structural integrity and functioning by mixing several materials with diverse mechanical properties [45-47].

Opportunities for real-time monitoring and feedback are made possible by the incorporation of sensors into 3D-printed cardiac implants. In order to personalize patient care and prompt intervention, sensors can give useful information on implant function, physiological parameters, and possible problems [48-50]. The selection of appropriate sensor types, their miniaturization, power supply, and compatibility with 3D-printed materials provide hurdles for the integration of sensors. Collaborations between materials scientists, engineers, and biological researchers are necessary to overcome these obstacles [2,51].

The development of patient-specific designs for 3D-printed cardiac implants depends heavily on medical imaging techniques like CT and MRI. These imaging techniques offer comprehensive anatomical details that help with the precise manufacture of implants specially made for each patient [31,52]. The performance of 3D-printed cardiac implants is increasingly being simulated using computational modeling approaches, such as finite element analysis. These models allow for the evaluation of the mechanical, hemodynamic, and long-term behavior of implants, resulting in improved designs and results [53,54].

Smart materials, such as shape memory alloys, hydrogels, and responsive polymers, can detect and react to outside stimuli. Smart materials can allow capabilities like self-regulation, medication release, and adaptive responses to physiological changes in 3D-printed cardiac implants [55]. Smart implants have the potential to revolutionize cardiac care by offering personalized therapies and enhanced patient outcomes. Drug-eluting 3D-printed stents are one example of how tailored medicines might be delivered to stop restenosis and encourage healing. Additionally, smart implants that adjust to the patient's physiological requirements might enhance the biocompatibility and long-term function of cardiac implants [56,57].

## Conclusions

Tremendous progress has been made in recent years in the development of 3D-printed heart valves and other cardiac implants. Improving customization, boosting biocompatibility, and maximizing functional performance are the current developments in the industry. In order to duplicate the complex structure and functionality of human heart valves, studies are investigating cutting-edge materials and manufacturing techniques. Additionally, imaging technologies for patient-specific design are being utilized, enabling the development of personalized models and specially made implants that exactly match different anatomical structures. Natural polymers with distinct benefits in terms of structural characteristics, biocompatibility, and tissue regeneration include collagen, fibrin, and chitosan. Biodegradability, biocompatibility, and mechanical strength are features of synthetic polymers such as PGA, PLLA, PLGA, poly(-caprolactone), and PU. Stem cells are essential to cardiac tissue engineering because they have the capacity to regenerate tissue and reduce immunological rejection. The accurate production of implants is made possible by CAD and CAM, and preoperative planning utilizing 3D-printed patient-specific anatomical models enhances surgical results. These advancements in 3D printing technology have the potential to transform cardiac healthcare by providing individualized and long-lasting solutions for patients with heart valve diseases and other cardiac conditions. To overcome the constraints and difficulties posed by these technologies, further investigation and research are required. Nonetheless, 3D-printed cardiac implants have the potential to completely revolutionize how cardiovascular disorders are treated in the future.
